# Shallow shotgun metagenomic sequencing of wild yeast communities enriched in ethanol-containing koji extract medium

**DOI:** 10.1128/mra.00346-26

**Published:** 2026-07-17

**Authors:** So Umekage

**Affiliations:** 1 Department of Food and Nutrition, Koriyama Women’s University, Koriyama Fukushima, Japan; University of California Riverside, Riverside, California, USA

**Keywords:** metagenome, yeast, koji

## Abstract

I report the shallow shotgun metagenomic sequencing data of three ethanol-enriched wild yeast communities cultured in an ethanol-containing koji extract medium.

## ANNOUNCEMENT

A koji extract-based enrichment medium prepared from *Aspergillus oryzae* has been used to isolate sake-brewing yeasts from environmental samples (1–5). To provide a basic resource for evaluating this enrichment strategy, shallow shotgun metagenomic sequencing data were obtained from wild yeast communities cultured in an ethanol-containing koji extract medium.

To obtain koji extract, amazake (a traditional Japanese fermented sweet drink) was first prepared. In brief, 150 g of commercially available raw rice koji (Houraiya, Koriyama, Japan) was incubated in 1 L of water at 55–58°C for 24 h, and the amazake was then filtered through ADVANTEC No. 2 filter paper (Toyo Roshi Kaisha, Ltd., Tokyo, Japan). Subsequently, sodium propionate [0.01% (wt/wt)] (Marugo Corporation, Soka, Japan), lactic acid [0.01% (vol/vol)], and chloramphenicol (20 µg/mL) (Fujifilm, Osaka, Japan) were added, and the mixture was autoclaved at 121°C for 15 min.

For wild yeast enrichment, 3.0 g of soil collected from a perilla field (located in Katsurao Village, Fukushima, Japan, 37.506893°N, 140.727705°E) and maintained by Koriyama Women’s University for educational purposes was added to 20 mL of koji extract in a 50 mL conical tube. The mixture was incubated under static conditions at 25°C, with the screw cap loosened and gently agitated once daily, until air bubble formation was observed. Then, 500 μL of the culture was added to each of three 20 mL portions of koji extracts containing 0, 3, and 5% ethanol. The cultures were incubated under static conditions at 25°C, with gentle agitation once daily, until the OD_660_ reached 1.6. The cells were harvested by centrifugation at 6,000**×** *g* for 2 min. Total DNA was extracted from the cells using the GenCheck DNA extraction reagent (FASMAC Co., Ltd., Kanagawa, Japan), and sequencing libraries were prepared by FASMAC Co., Ltd. using the KAPA EvoPlus V2 Kit (Roche, Pleasanton, CA, USA) according to the manufacturer’s instructions. Sequencing was performed on a DNBSEQ-G400 platform with an FCL flow cell (MGI Tech, Shenzhen, China).

Raw paired-end FASTQ files were assessed using FastQC (v0.12.1) (6). The 0, 3, and 5% ethanol-containing samples yielded 5,522,823, 5,423,337, and 5,375,185 read pairs, respectively. The corresponding read-length ranges were 8–150, 9–150, and 3–150 bp, and the guanine and cytosine (GC) contents were 33.0, 32.5, and 35.0%, respectively (Table 1). For all samples, the per-base sequence quality and adapter content passed quality control. Reads that mapped to the human reference genome GRCh38/hg38 were removed using Bowtie2 (v2.5.4) (7), and the remaining reads were analyzed using Kraken2 (v2.17.1+galaxy0) (8) and Bracken (v3.1+galaxy0) (9) on the Galaxy platform. Default parameters were used, except where otherwise noted. The Kraken2 confidence threshold was set to 0.05, and the PlusPF database was used for taxonomic classification. *Torulaspora delbrueckii* was the predominant species in the 3 and 5% ethanol samples.

**TABLE 1:** Summary statistics of the metagenomic sequencing reads^*a*^

Ethanol (%)	BioSample accession	Run accession	Read pairs	Bases (Mbp)	Read length range (bp)	GC (%)	Per-base sequence quality	Adapter content
0	SMAD01830183	DRR915747	5,522,823	812.5	8–150	33.0	PASS	PASS
3	SMAD01830184	DRR915748	5,423,337	801.5	9–150	32.5	PASS	PASS
5	SMAD01830185	DRR915749	5,375,185	791.5	3–150	35.0	PASS	PASS

^*a*^ GC content is shown as the average of the R1 and R2 reads.

## Data Availability

The shallow shotgun metagenomic sequencing data were deposited in the DNA Data Bank of Japan (DDBJ) Sequence Read Archive (DRA) under BioProject accession number PRJDB40616. The DRA run accession numbers are DRR915747, DRR915748, and DRR915749.
